# Study of the Long-Term Results of Decompressive Craniectomy after Severe Traumatic Brain Injury Based on a Series of 60 Consecutive Cases

**DOI:** 10.1155/2014/207585

**Published:** 2014-02-24

**Authors:** Gaétane Gouello, Olivier Hamel, Karim Asehnoune, Eric Bord, Roger Robert, Kevin Buffenoir

**Affiliations:** ^1^Service de Neurotraumatologie et Neurochirurgie, CHU de Nantes, 44093 Nantes, France; ^2^Service d'Anesthésie Réanimation Chirurgicale, CHU de Nantes, 44093 Nantes, France; ^3^INSERM EA3826, “Pain, Neuromodulation, and Quality of Life”, CHU de Nantes, 44093 Nantes, France

## Abstract

*Background*. Decompressive craniectomy can be proposed in the management of severe traumatic brain injury. Current studies report mixed results, preventing any clear conclusions on the place of decompressive craniectomy in traumatology. *Methods*. The objective of this retrospective study was to evaluate the results of all decompressive craniectomies performed between 2005 and 2011 for refractory intracranial hypertension after severe traumatic brain injury. Sixty patients were included. Clinical parameters (Glasgow scale, pupillary examination) and radiological findings (Marshall CT scale) were analysed. Complications, clinical outcome, and early and long-term Glasgow Outcome Scale (GOS) were evaluated after surgery. Finally, the predictive value of preoperative parameters to guide the clinician's decision to perform craniectomy was studied. *Results*. Craniectomy was unilateral in 58 cases and the mean bone flap area was 100 cm^2^. Surgical complications were observed in 6.7% of cases. Mean followup was 30 months and a favourable outcome was obtained in 50% of cases. The initial Glasgow Scale was the only statistically significant predictive factor for long-term outcome. *Conclusion*. Despite the discordant results in the literature, this study demonstrates that decompressive craniectomy is useful for the management of refractory intracranial hypertension after severe traumatic brain injury.

## 1. Introduction

The incidence of traumatic brain injury (TBI), regardless of severity, is difficult to evaluate. North American data indicate an estimated incidence of about 500/100,000 inhabitants, requiring hospitalisation in 20% of cases and leading to death in 3% of cases [1]. The incidence of severe traumatic brain injury is decreasing in developed countries but is rapidly growing in emerging countries. It has been estimated that, in the medium term, a member of one in every 200 families will be a victim of severe TBI in these countries (Brazil, Argentina, China, and India). In Europe, 8,000 people each year die from TBI. Management of these patients remains difficult, both in terms of management of the acute phase and in terms of the human and socioeconomic burden. A North American cost-benefit analysis conducted in 2007 evaluated the mean cost of management of each TBI patient. Costs related to acute care, rehabilitation, and loss of productivity for society were estimated to be $60,887, $4,618, and $33,087 per patient, respectively [2]. Management of severe TBI therefore remains a major public health problem at the present time.

The combined medical and surgical objective during the acute phase is to prevent intracranial hypertension and maintain satisfactory cerebral perfusion pressure (CPP) in order to limit the development of secondary lesions. Some surgical procedures designed to achieve this objective are consensual, such as evacuation of a compressive extra-axial haematoma. The modalities of surveillance of severe TBI have evolved, but the gold standard currently remains combined monitoring of intracranial pressure (ICP) and mean arterial pressure (MAP) [3–5]. Medical treatments initiated during management of severe TBI are defined according to detailed guidelines published by the Brain Trauma Foundation (BTF) [6]. Decompressive craniectomy may need to be considered when the situation is no longer controlled by these techniques.

In 1901, Kocher reported the use of the large craniectomy for the treatment of posttraumatic cerebral oedema refractory to medical treatment [7]. This technique was subsequently abandoned due to the high postoperative complication rate but is now widely used and has been the subject of numerous published series [8–21]. However, the indications for decompressive craniectomy remain difficult to define for the surgeon in the emergency setting, as this technique remains a controversial issue in the literature. The main parameter to be taken into account is not the survival rate after craniectomy, but the functional outcome of these patients. In this study, we report the outcome with a followup of more than 2 years in a series of 60 consecutive patients with severe TBI, in whom an indication for decompressive craniectomy was adopted in our institution between 2005 and 2011.

## 2. Material and Methods

### 2.1. Study Description

This single-centre, retrospective, descriptive study was based on a patient cohort managed by decompressive craniectomy in the context of severe TBI between January 2005 and May 2011. As this was a retrospective, observational study, according to French legislation (article L.1121-1 paragraph 1 and R1121-2 of the French Public Health Code), neither informed consent nor ethics committee approval was required to use data for an epidemiological study.

#### 2.1.1. Inclusion Criteria

All patients undergoing unilateral or bilateral decompressive craniectomy following head injury during this period were included in the study. This study included patients operated on after failure of medical treatment with ICP monitoring, as well as patients operated on immediately because of a surgical posttraumatic lesion, in whom ICP monitoring was not performed before surgery and in whom the bone flap was not repositioned due to the presence of cerebral oedema or major intraoperative brain swelling.

#### 2.1.2. Exclusion Criteria

Patients managed in the operating room who presented bilateral fixed dilated pupils and absence of oculocardiac reflex were excluded.

### 2.2. Data Collection

Data collection was performed retrospectively from review of medical charts and a database. Patients were recruited from this database on the basis of coding of surgical procedures and diagnoses, allowing the inclusion of all patients undergoing decompressive craniectomy for severe TBI during the study period.

All cases were reviewed on the basis of SMUR (paramedics) intervention reports, analysis of all brain imaging examinations, laboratory test results, operation reports, intensive care nursing records, intensive care, neurotraumatology and rehabilitation discharge summaries, and outpatient visit reports. Peripheral centres, local rehabilitation centres, and general practitioners were also contacted to obtain missing data, especially concerning the long-term outcome or for patients living a long way from the hospital. Data were anonymised and entered into an Excel database (Microsoft Office Excel 2003 SP3).

#### 2.2.1. Demographic Data

The patient's age, gender, history (previous use of platelet antiaggregants or anticoagulants), the mode of injury, and associated injuries were recorded.

#### 2.2.2. Prehospital and Hospital Data

The initial Glasgow score, the presence of unilateral or bilateral dilated pupils, the presence of a corneal reflex in the case of bilateral dilated pupils, and the various treatments administered before craniectomy, such as mannitol or barbiturates, were recorded. Finally, ICP and CPP values during management were analysed.

#### 2.2.3. Imaging

All initial imaging examinations were reviewed with description of the lesions, measurement of midline shift, and classification of lesions according to Marshall's classification [22]. Bone flap areas and largest bone flap diameter for each patient were calculated from a postoperative thin-slice CT scan using Carestream PACS version 11.0 imaging software (Carestream Health).

#### 2.2.4. Postoperative Data

Postoperative complications were systematically recorded. The number of days spent in the intensive care unit and in conventional wards was calculated. The Glasgow Outcome Scale [23] was calculated for each patient 3 months after the operation and at long-term followup.

### 2.3. Medical Treatment

Patients were sedated with a continuous intravenous infusion of fentanyl (2 to 5 *μ*g·kg^−1^·hr^−1^) and midazolam (0.2 to 0.5 mg·kg^−1^·hr^−1^). Secondary brain injuries were prevented by keeping the body temperature between 36.0°C and 37.0°C, ensuring normoglycemia and normocapnia, and by avoiding hypoxia. Serum sodium and arterial blood gases were assessed at least twice daily and expiratory end-tidal (Et) CO_2_ was continuously monitored. Patients were monitored by invasive arterial pressure measurement and mean arterial pressure was measured up to the brain for calculation of CPP. In patients with severe TBI and abnormal computed tomography (haematomas, contusions, swelling, herniation, or compressed basal cisterns), ICP was monitored by an intraparenchymal probe placed in the more severely affected side (Codman, Johnson and Johnson Company, Raynham, MY, USA). CPP was maintained >65 mmHg with isotonic saline (0.9% NaCl) and vasopressor (norepinephrine). Mannitol (0.5 g/kg bolus, repeated once in the case of poor ICP control (ICP > 20 mmHg) after 30 minutes; maximum dose, 1 g/kg) was used to control episodes of ICH. In the case of poor ICH control, midazolam infusion was continued, and barbiturate (sodium thiopental) was used (loading dose of 2 to 3 mg/kg) followed by continuous infusion (starting dose of 2 to 3 mg/kg/hr) adapted to the course of ICP and, once a day, to monitoring of serum thiopental levels (target: 20 to 30 *μ*g/mL).

### 2.4. Surgical Technique

The frontotemporoparietal skin incision was shaped like a question mark or crossbow. Crossbow incisions were associated with poor tissue viability at the junction, with a risk of necrosis. A large flap base was required. The rules of blood supply and innervation must be observed for such incisions. Briefly, the incision must spare the superficial temporal artery and especially the temporal branch of the facial nerve, ensuring motor innervation of the upper part of the face and which, in most studies, crosses the zygomatic arch 1 cm anteriorly to the tragus. This position of the temporal branch of the facial nerve has been extensively described in the literature with several variants in relation to the zygomatic arch [24].

A very large bone flap was created with the craniotome via several burr holes. To create a frontotemporoparietal bone flap, it is recommended to follow the superior sagittal sinus and raise the frontal bone as far as the frontal sinuses, temporal bone, and parietal bone as far as the lateral sinus posteriorly. As far as possible, the bone flap must extend as far as the skull base in the temporal bone while avoiding creating other cerebral parenchymal lesions at this zone as a result of brain swelling.

The dura mater was then coagulated and widely opened, either by means of a cross-shaped incision or by several divisions. A duraplasty was performed either by a periosteal patch or by synthetic material using sheets of Surgicel Nu-Knit or Neuropatch. Periosteum was simply replaced without sutures, and subcutaneous and skin sutures were then performed. A Redon suction drain was sometimes placed subcutaneously for 48 hours when considered necessary by the surgeon.

### 2.5. Statistical Analysis

Statistical analysis was performed with Excel software (Microsoft Office Excel 2003 SP3) and online statistical software Open Source R version 2.14.1 (the R project for statistical computing). Patient characteristics were expressed as the median, mean, and standard deviation (SD). Quantitative variables were expressed as the mean, standard deviation and *P* value and qualitative variables were expressed as number, percentage, and *P* value with a limit of significance to 0.05 (*α* risk of 5%). A normal distribution was tested for each variable. Prognostic factors (age, history, initial Glasgow score, presence of pupillary abnormalities, time to management, size of the bone flap, and initial imaging findings) were studied separately on univariate analysis. Parametric tests (Chi^2^ test) were used for quantitative variables and nonparametric tests (Chi^2^ test) were used for qualitative variables (Welch's *t*-test or Mann-Whitney test) taking into account the small sample size of the population.

## 3. Results

### 3.1. Demographic Data

**Figure 1:**
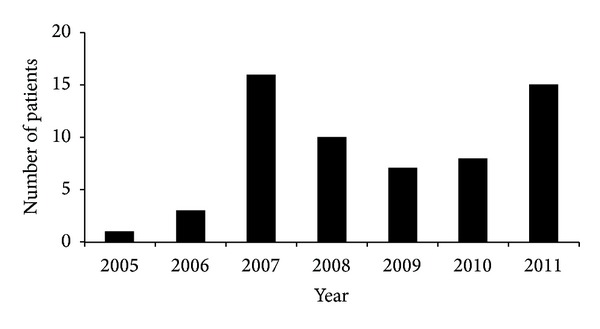
Distribution of the number of decompressive craniectomies performed each year between 2005 and 2011.

Sixty patients were included.  Figure 1 shows the number of patients included each year. On the basis of preliminary data analysis, patients were divided into two groups according to the indication for decompressive craniectomy:

- the first group (group 1) of 40 patients corresponded to the group of patients initially treated by the best medical treatment and operated on in a context of refractory intracranial hypertension,
- the second group (group 2) of 20 patients corresponded to patients who were operated on immediately for evacuation of compressive haematoma, in whom decompressive craniectomy was also performed in the light of intraoperative findings (episode of severe oedema, making it impossible to replace the bone flap).

All demographic data for these two groups are presented in Table 1. The mean age was 33 years (range: 2–64 years) with a very marked male predominance: 77% of men. Only one patient had a major medical history with platelet aggregation inhibitor and Kardégic (aspirin) therapy. No patients were treated with oral anticoagulants, and 43% of patients had a positive blood alcohol level higher than the legal limit of 0.5 g/L at the time of initial management. The two groups can be considered to be comparable in terms of all demographic variables (see statistical data in Table 1).

### 3.2. Prehospital and Hospital Data

#### 3.2.1. Initial Glasgow Score

The mean Glasgow score at the time of initial management for the two groups combined was 7.23, with no significant difference between the two groups (Table 1). All patients with a Glasgow score higher than 8 deteriorated over the first 3 hours following the trauma with a subsequent score less than or equal to 7, corresponding to severe TBI.

#### 3.2.2. Pupillary Abnormalities

Twenty-six patients (43%) presented symmetrical constricted pupils and 34 patients (57%) presented dilated pupils, bilateral in 13 cases. The corneal reflex was tested in patients with bilateral dilated pupils and was absent in 6 patients. The oculocardiac reflex was present in all cases. These results are presented in Table 1. A significantly higher proportion of patients with dilated pupils were observed in group 2, due to the presence of compressive haematomas requiring surgical evacuation in this group.

#### 3.2.3. Medical Treatment and ICP

All patients of group 1 were initially managed in the intensive care unit for monitoring of ICP and CPP and medical treatment as a function of the results of this monitoring, according to guidelines [6]. This treatment comprised optimization of sedation with nonbarbiturate anaesthetics, osmotherapy (including controlled hypernatraemia), and maintenance of MAP by means of norepinephrine. The treatment target was to maintain ICP less than 20 mmHg and CPP greater than 65 mmHg. This treatment was maintained and optimized during the first twelve hours. When patients remained refractory to this treatment (ICP > 20 mmHg and CPP < 65 mmHg) after the first 12 hours, decompressive craniectomy was considered before initiating barbiturate anaesthetics.

ICP and CPP were not monitored preoperatively in patients of group 2, who were immediately transferred to the operating room for evacuation of a compressive subdural haematoma.

### 3.3. Imaging Findings

The distribution of imaging findings according to the Marshall score is described in Table 1. Group 2 presented higher grade radiological lesions according to this classification (*P* = 0.003), which can be explained by the fact that these patients of group 2 initially presented compressive lesions for which immediate surgery was indicated. All of these patients presented an acute subdural haematoma classified as “evacuated mass” when it was estimated to be larger than 25 cc and as Marshall grade II in the other 4 cases.

### 3.4. Surgical Findings

The mean time to surgical management, that is, between the head injury and decompressive craniectomy, was 1.75 days (Table 1). Group 1 comprised patients operated on after 24 hours and group 2 comprised patients operated on during the first 24 hours (compressive acute subdural haematoma).

Unilateral craniectomy, on the side contralateral to the midline shift, was performed in 58 patients structures and bifrontal craniectomy was performed in 2 patients with diffuse cerebral oedema with no midline shift.

The size of the bone flap was estimated on postoperative brain CT scans, with measurement of the bone flap largest diameter and bone flap area. The size of the bone flap could not be measured in four patients who died prior to postoperative imaging. The mean bone flap diameter was 12 cm (range: 7.5–15 cm) and the mean bone flap area was 100 cm^2^ (range: 32–132 cm^2^). No significant difference was observed between the sizes of the bone flaps in the two groups of patients (Table 2). Note that the smallest flap (32 cm^2^) was performed in a 2-year-old child following evacuation of an acute subdural haematoma, in whom a severe episode of cerebral oedema is major contraindicated closure by the bone flap.

### 3.5. Postoperative Course

#### 3.5.1. Postoperative Complications

Four patients developed hydrocephalus requiring cerebrospinal fluid shunt placement. This complication was related to initial intraventricular bleeding and was therefore not due to the craniectomy procedure. Four complications attributed to the surgical procedure were observed, corresponding to a complication rate of 6.7%: two wound infections requiring surgical revision with a satisfactory outcome and two wound haematomas (one extradural haematoma and one subdural haematoma) also requiring surgical revision, with no further complications.

#### 3.5.2. Duration of Followup

Seventeen deaths occurred during the first postoperative month. These patients were excluded from calculation of length of stay and followup. The mean length of stay in the intensive care unit was 36.6 days (range: 7–120 days) and the mean length of stay in conventional wards was 32 days (range: 0–240 days). The mean followup, corresponding to the interval between initial management and last functional assessment, was 30.7 months (range: 24–78 months).

#### 3.5.3. Functional Outcome

The GOS was evaluated three months postoperatively, then more than 24 months postoperatively. The results are presented in Table 2. Analysis of these data shows an overall mortality rate of 28.3%, which was much higher in the group of patients in whom surgery was performed immediately (group 2). The outcome of survivors was classified as unfavourable (GOS 2 and 3) or favourable (GOS 4 and 5). Functional improvement continued over time for patients of group 1 (favourable outcome improved from 42.5% to 60% of patients) but remained identical between the early assessment and the long-term assessment for patients of group 2. While no significant difference was observed between groups 1 and 2 at 3 months (Welch's *t*-test, *P* = 0.061), a significantly better outcome was demonstrated for patients of group 1 compared to patients of group 2 at the long-term assessment (Welch's *t*-test, *P* = 0.012).

### 3.6. Prognostic Factors

Prognostic factors for mortality were investigated by comparing the populations of deceased patients and survivors in terms of the following variables: age, gender, presence of pupillary abnormalities, initial Glasgow score, Marshall classification, time to surgery, and bone flap area. The results are presented in Table 3. Univariate analysis showed that pupillary abnormalities (Welch's *t*-test, *P* = 0.003) and the initial Glasgow score (Welch's *t*-test, *P* = 0.014) were significantly associated with mortality. This study therefore shows that the presence of bilateral dilated pupils and a Glasgow score less than or equal to 5 are high-risk factors for mortality.

Prognostic factors of favourable long-term functional outcome were also investigated by comparing the GOS 2 and 3 group to the GOS 4 and 5 group. The results are presented in Table 4. Univariate analysis showed that only the initial Glasgow score was significantly correlated with functional outcome (Welch's *t*-test, *P* = 0.048). In contrast, no statistically significant association was demonstrated between functional outcome and age, gender, presence of pupillary abnormalities, initial imaging, time to surgery, and bone flap area.

Identification of prognostic factors was also performed separately on the two groups of patients (groups 1 and 2). This analysis did not identify any other prognostic factors and is therefore not presented here.

## 4. Discussion

### 4.1. Should Decompressive Craniectomy Be Performed in Severe Traumatic Brain Injury?

Decompressive craniectomy in the context of severe TBI has been the subject of numerous publications over the last twenty years with variable results. Most of these studies presented a low level of proof and were retrospective studies based on small series of patients. Variable results have been reported, as these studies were based on different populations in terms of age, exclusion criteria, and operative techniques. It is therefore difficult to compare the results of these studies, but the long-term results of several published series as well as those of the present series are summarized in Table 5.

The Cochrane Collaboration conducted a review of the literature in 2009 [25], based on 154 studies. This review established the benefit of decompressive craniectomy in terms of a reduction of the mortality rate and a reduction of the risk of long-term unfavourable results for only one randomized study conducted in children [21]. However, this review of the literature comprising clinical case reports and nonrandomized retrospective and prospective studies suggested a real benefit of decompressive craniectomy in patients with severe TBI.

The DECRA protocol (decompressive craniectomy) [12] was a multicentre, randomized, controlled study in adults under the age of 60 years comprising 155 randomized patients, 73 of whom were operated on. This study demonstrated a benefit in terms of reduction of postoperative ICP but did not demonstrate any significant differences between the groups of operated and nonoperated patients. Favourable long-term results were observed in only 30% of craniectomized patients. However, the conclusions of this study have been questioned by a number of authors [26–28], due to the bias related to the absence of equivalence between the two groups with more patients presenting bilateral dilated pupils in the group of operated patients. The choice of operative technique, comprising bilateral (bifrontotemporoparietal) craniectomy, was also controversial, as this technique is associated with a higher complication rate. This study was also based on a small sample size, representing only a limited subsample of patients with traumatic brain injury, as, in particular, patients with cerebral contusion were not included in the study. Some authors have also questioned the inclusion criteria of this study, considering that the ICP value adopted for randomization was too low (ICP > 20 mmHg for more than 15 minutes). Finally, the interval between the head injury and randomization, and consequently surgery, was also too long (an average of 32 hours). In view of these various criticisms, although this study was prospective and randomized, it cannot be considered to have clearly resolved the question of the benefit of craniectomy in severe TBI.

Published results on long-term functional outcome evaluated by GOS are very heterogeneous with favourable results ranging from 19% to 71.5% (Table 5), but it is difficult to compare these results, as these studies were conducted in very different populations with various exclusion criteria: age, but especially the presence of pupillary abnormalities. For example, some series excluded patients with bilateral dilated pupils and therefore reported better results. These series also did not specify whether craniectomy was systematically excluded in all patients with bilateral dilated pupils or whether craniectomy was performed and these patients were subsequently excluded from the study. In contrast, the present series comprised all decompressive craniectomies performed in our unit, regardless of the initial clinical state.

It is difficult to reliably compare the various published series due to the variable severity of head injuries (initial Glasgow score). Furthermore, the Glasgow score is also difficult to compare from one study to another, as, although some studies like our own are based on the Glasgow score evaluated at the time of initial management, other studies are based on the Glasgow score evaluated at the patient's arrival in the centre or that evaluated before intubation.

Finally, operative techniques also differ from one study to another, as, in the series reported by Cooper et al. [12], only bilateral craniectomies were performed. Similarly, the series by Ecker et al. [14] reported the results for 33 patients operated on by bifrontal, bihemispheric or supratentorial, and infratentorial craniectomies, while a total of 188 patients underwent craniectomy during the study period. In the present series, 58 of the 60 patients underwent unilateral craniectomy. In our institution, the choice of technique is based on the initial imaging findings. Bilateral craniectomy was only performed in our series in the presence of diffuse oedema with no midline shift.

In the light of these discordant results of the literature and the favourable results obtained in group 1 of the present series (60% of GOS 4 and 5 after more than 24 months), we believe that decompressive craniectomy should be considered in the management of severe traumatic brain injury. The essential problem concerns the definition of prognostic factors.

### 4.2. Prognostic Factors

The present study demonstrated an association between an initial Glasgow score greater than 8 and a favourable functional outcome. In contrast, an initial Glasgow score less than 5 was associated with a statistically higher mortality rate. Nevertheless, some studies have reported a strong correlation between the Glasgow score and functional outcome [11, 29].

In our series, the presence of bilateral fixed dilated pupils was the only other prognostic factor associated with excess mortality.

Considerable differences in mean age were observed between the various published series (few paediatric series, but also few elderly patients included). Some series excluded patients over the age of 50 years, as in the study by Guerra et al. [15], which reported very favourable results but based on a younger population. The paediatric series published by Taylor et al. [21] also reported good results. The study by Ecker et al. [14], which also reported good results, only included young patients with a mean age of 24 years, essentially composed of soldiers with no medical history and in very good physical condition. The present study, including patients of all age groups (8 children under the age of 15 years and 13 patients at the age of 50 years and older), did not demonstrate any correlation between age and prognosis (mortality or functional outcome). In our series, the outcome, for an equivalent initial Glasgow score, appeared to be better in patients older than 50 (1 GOS 1, 2 GOS 3 and 4, and 10 GOS 4 and 5) than in children under the age of 15 years (2 GOS 1, 4 GOS 3 and 4, and 2 GOS 4 and 5). However, other series, especially that published by Chibbaro and Tacconi [11], have demonstrated a significant association between young age and long-term functional outcome. Similarly, Polin et al. [19] also reported statistically better results in children. The patient's age therefore does not appear to be a reliable prognostic factor at the present time.

The time to craniectomy also did not appear to be a prognostic factor in our series. Some studies have analysed the relationship between time to surgery and mortality. The study by Faleiro et al. [30] reported a higher mortality rate in the early surgery group than when surgery was performed after the 24th hour (59% versus 53%). Wen et al. [31] showed that early craniectomy did not improve the functional outcome. Unfortunately, continuous ICP monitoring was not performed in our study, but our clinical impression is that patients with ICP higher than 40–50 mmHg at the initial assessment and in whom medical treatment remains ineffective have a poor prognosis (GOS 1 or 2) after craniectomy. Those patients in whom medical treatment is initially effective but who subsequently present raised ICP after several hours tend to have a favourable outcome after craniectomy (GOS > 3). However, this distinction requires a more detailed analysis of ICP curves at the initial phase of trauma.

The size of the bone flap appears to be an important element, as the larger the bone flap, the lower the risk of parenchymal injury at the bone margins. This point has been highlighted by several authors [12]. However, no significant correlation was observed between the size of the bone flap and overall functional prognosis in our series.

The Marshall score for the initial imaging findings did not appear to be a prognostic factor for outcome in our study. Lesions are difficult to evaluate on CT scan at the initial phase of the trauma. Some authors have reported the value of MRI at the early phase of trauma, particularly diffusion-weighted sequences [32] and metabolic imaging techniques [33]. These techniques are very probably useful to guide the decision of whether or not to perform craniectomy, but their role needs to be confirmed by further studies.

Finally, other prognostic factors have been reported in the literature, such as biomarkers, particularly protein S100. Bouvier et al. [34] showed that high levels of protein S100 at initial management were associated with poor prognosis. However, the value of this marker on long-term outcome needs to be assessed in prospective studies.

## 5. Conclusion

Overall, in the patient cohort presented here (60 consecutive decompressive craniectomies performed in the context of severe TBI between 2005 and 2011), a favourable functional outcome was observed in 50% of cases. A higher rate (60%) of favourable functional outcome was observed for patients with a higher Glasgow score at the time of initial management, receiving the best medical care to prevent intracranial hypertension, and who subsequently become refractory to these resuscitation measures.

The results of this study therefore encourage us to perform fewer immediate decompressive craniectomies in patients with a very severe traumatic brain injury (initial Glasgow score <5 and bilateral fixed dilated pupils). In contrast, in patients with an initial Glasgow score greater than 7, who deteriorate over the first hours after admission or in whom ICP is initially well controlled by intensive care (excluding barbiturates), but who subsequently become refractory to these treatments, decompressive craniectomy remains a useful treatment option. The decision to perform decompressive craniectomy remains more difficult in intermediate situations and must be based on a case-by-case multidisciplinary review.

## Figures and Tables

**Table 1:** Patient characteristics in group 1 and group 2.

	Group 1		Group 2		Total			*P* value
	*n* = 40		*n* = 20		* n* = 60			
	Number	Mean	Number	Mean	Number	Mean	%	
Age (years)								
≤15 years	5	31.5	3	33.5	8	33.0	13.3	0.800^*ω*^
16–30 years	14		7		21		35.0	
>30 years	21		10		31		51.7	
Gender								
Male	31	NA	15	NA	46	NA	76.7	0.910^*ψ*^
Female	9		5		14		23.3	
Initial Glasgow score								
3 to 5	15	7.22	11	7.25	26	7.23	43.4	0.540^*ω*^
6 to 8	14		3		17		28.3	
>8	11		6		17		28.3	
Preoperative pupillary abnormality								
No	23	NA	3	NA	26	NA	43.3	**0.031** ^*ψ*^
Unilateral dilated	11		10		21		35	
Bilateral dilated (absent corneal reflex)	6 (2)		7 (4)		13 (5)		21.7 (8.3)	
Multiple trauma								
Yes	17	NA	9	NA	26	NA	43.3	0.930^*χ*^
No	23		11		34		56.7	
Circumstances of trauma								
Road accident	26	NA	10	NA	36	NA	60.0	NA
Fall from a great height	3		5		7		11.7	
Fall from a small height	11		5		17		28.3	
Initial CT analysis of the lesions according to the Marshall classification								
I	14	NA	0	NA	14	NA	23.3	**0.003** ^*ω*^
II	12		0		12		20.0	
I + II	**26**		**0**		**26**		**43.3**	
III	1		0		1		1.7	
IV	13		4		17		28.3	
III + IV	**14**		**4**		**18**		**30.0**	
Evacuated mass	**0**		**16**		**16**		**26.7**	
Interval between trauma and decompressive craniectomy								
<6 h	4	2.35 days	16	0.55 days	20	1.75 days	33.3	**<0.001** ^*ω*^
6 h to 24 h	7		0		7		11.7	
24 h to 7 days	26		4		30		50.0	
>7 days	3		0		3		5.0	

Preoperative characteristics of patients in group 1 and group 2 are expressed by number in each category and the mean value or percentage with respect to the total sample size (%). Values for the 2 groups were compared by statistical tests evaluating the *P* value (*α* < 5%).

^*ω*^Welch *t*-test; ^*ψ*^Mann-Whitney test; ^*χ*^Chi-square test.

MVA: motor vehicle accident; VKA: vitamin K antagonist; PAI: platelet aggregation inhibitor; NA: not applicable.

The bold font highlights the most significant parameters.

**Table 2:** Functional outcome of the patients evaluated by the Glasgow Outcome Scale (GOS) 3 months and more than 24 months after decompressive craniectomy.

	Group 1		Group 2		Total		*P* value
	*n* = 40		*n* = 20		*n* = 60		
	Number	%	Number	%	Number	%	
GOS at 3 months							
1	7		10		17		
2	5		1		6		
3	11		3		14		
4	11		3		14		
5	6		3		9		
Death (1)	**7**	**17.5**	**10**	**50.0**	**17**	**28.3**	0.061
Unfavourable (2 + 3)	**16**	**40.0**	**4**	**20.0**	**20**	**33.3**	
Favourable (4 + 5)	**17**	**42.5**	**6**	**30.0**	**23**	**38.4**	
GOS after 24 months							
1	7		10		17		
2	5		1		6		
3	4		3		7		
4	14		2		16		
5	10		4		14		
Death (1)	**7**	**17.5**	**10**	**50.0**	**17**	**28.3**	**0.012**
Unfavourable (2 + 3)	**9**	**22.5**	**4**	**20.0**	**13**	**21.7**	
Favourable (4 +5)	**24**	**60.0**	**6**	**30.0**	**30**	**50.0**	

The *P* value column corresponds to the comparison of the distribution of functional outcome between the two patient groups (Welch's *t*-test).

**Table 3:** Analysis of prognostic factors for mortality.

	Deceased patients	Surviving patients	*P* value
	*n* = 17	*n* = 43	
Age			
<15 years	4	4	0.097^*ω*^
15 to 30 years	7	14	
>30 years	6	25	
Gender			
Male	13	33	0.750^*χ*^
Female	4	10	
Pupillary examination			
Constricted	3	23	**0.003** ^*ω*^
Unilateral dilated	6	15	
Bilateral dilated (absent corneal reflex)	8 (4)	5 (2)	
Initial Glasgow score			
3 to 5	13	13	**0.014** ^*ω*^
6 to 8	2	15	
>8	2	15	
mean	4.3	8.0	
Time to surgery			
<6 h	7	13	0.183^*ω*^
6 h–24 h	3	4	
24 h–7 days	7	23	
>7 days	0	3	
Bone flap area (cm^2^) (mean ± SD)	103.2 ± 24.2	98.7 ± 21.3	0.555^*ω*^
Marshall score			
I	2	12	0.403^*ω*^
II	3	9	
III	0	1	
IV	4	13	
Evacuated mass	8	8	

Comparison of the group of deceased patients and the group of surviving patients as a function of various prognostic criteria. A criterion was considered to have a prognostic value when a statistically significant difference was observed between the 2 groups with *P* < 0.05 (*α* = 5%).

^*ω*^Welch's *t*-test; ^*χ*^Chi^2^ test.

**Table 4:** Analysis of prognostic factors for functional outcome.

	GOS 2 + 3	GOS 4 + 5	*P* value
	*n* = 13	*n* = 30	
Age			
<15 years	2	2	0.125^*ω*^
15 to 30 years	6	8	
>30 years	5	20	
Gender			
Male	10	23	0.706^*χ*^
Female	3	7	
Pupillary examination			
Constricted	6	17	0.310^*ω*^
Unilateral dilated	4	11	
Bilateral dilated (absent corneal reflex)	3 (0)	2 (1)	
Initial Glasgow score			
3 to 5	7	6	**0.048** ^*ω*^
6 to 8	3	12	
>8	3	12	
Mean	6.2	8.8	
Time to surgery			
<6 h	5	8	0.800^*ω*^
6 h–24 h	1	3	
24 h–7 days	5	18	
>7 days	2	1	
Bone flap area (cm^2^) (mean ± SD)	101.1 ± 25.2	99.3 ± 19.6	0.926^*ω*^
Marshall score			
I	2	10	0.500^*ω*^
II	4	5	
III	0	1	
IV	2	11	
Evacuated mass	5	3	

Comparison of the group of patients with an unfavourable functional outcome (GOS 2 and 3) and the group of patients with a favourable functional outcome (GOS 4 and 5) as a function of various prognostic criteria. A criterion was considered to have a prognostic value when a statistically significant difference was observed between the 2 groups with *P* < 0.05 (*α* = 5%).

^*ω*^Welch's *t*-test; ^*χ*^Chi^2^ test.

**Table 5:** Review of the literature presenting the various series of decompressive craniectomies performed in the setting of severe traumatic brain injury.

Author, year	*n*	Age	GCS	Rand	GOS 4-5	GOS 2-3	GOS 1
Kjellberg and Prieto Jr., 1971 [17]	73	3 months–84 years	NR	No	NR	NR	72.0%
Polin et al., 1997 [19]	35	18.7 years	5.62	No	37.0%	40.0%	23.0%
Guerra et al., 1999 [15]	57	<50 years	NR	No	58.0%	20.0%	19.0%
De Luca et al., 2000 [13]	22	NR	NR	No	41.0%	41.0%	18.0%
Taylor et al., 2001 [21]	13	121 months	5 ± 2	**Yes**	54.0%	NR	NR
Schneider et al., 2002 [20]	62	36.6 years	6	No	29.1%	48.4%	22.5%
Albanèse et al., 2003 [9]	27	32 ± 15 years	5 ± 2	No	19%	30%	52%
Aarabi et al., 2006 [8]	50	25.3 years	7	No	40.0%	32.0%	28.0%
Chibbaro and Tacconi, 2007 [11]	48	47 [18–66] years	7	No	40.0%	45.0%	15.0%
Olivecrona et al., 2007 [18]	21	39.1 years	6.5	No	71.5%	11.4%	14.3%
Ho et al., 2008 [16]	16	38 [20–72] years	5 [3–7]	No	31%	31.5%	37.5%
Bao et al., 2010 [10]	37	NR	NR	No	54.1%	27.0%	18.9%
Cooper et al., 2011 [12]	73	<60 years	NR	**Yes**	30.0%	51.0%	19.0%
Ecker et al., 2011 [14]	33	24 [19–46] years	5 [3–14]	No	60.0%	17.0%	23.0%

Our series 2013	60	33 [2–64] years	7.23	No	**50.0%**	21.7%	28.3%

*n*: number of patients; GCS: mean initial Glasgow Coma Scale score; Rand: randomization; GOS: Glasgow Outcome Scale; ±*x*: standard deviation; [*x*–*y*]: range.
